# Favorable clinical outcomes are achieved in both male and female following medial meniscus posterior root repair

**DOI:** 10.1007/s00590-025-04344-y

**Published:** 2025-06-14

**Authors:** Haruyoshi Katayama, Takayuki Furumatsu, Yuki Okazaki, Naohiro Higashihara, Yusuke Yokoyama, Masanori Tamura, Koki Kawada, Tsubasa Hasegawa, Toshiki Kohara, Toshifumi Ozaki

**Affiliations:** 1 Okayama University Hospital, Okayama, Japan; 2 Okayama Red Cross General Hospital, Okayama, Japan

**Keywords:** Clinical outcome, Medial meniscus, Posterior root tear, Pullout repair, Sex difference

## Abstract

**Purpose:**

In recent years, medial meniscus (MM) posterior root tears (PRT) have received increasing attention due to their association with rapidly progressive knee osteoarthritis. MM posterior root (PR) repair has been reported to yield good clinical outcomes, but no study has yet to compare the postoperative outcomes after MMPR repair between sexes. The purpose of this study is evaluating the postoperative clinical outcomes following MMPR pullout repair by sex.

**Methods:**

Eighty-six patients who underwent pullout repair for isolated MMPRTs at our institution between October 2016 and November 2019 were evaluated. Patients were divided into two groups according to sex, and their clinical outcomes were compared preoperatively and at 2 years postoperatively.

**Results:**

The cohort was comprised of 21 male and 65 female patients. Three factors related to physical status (height (*p* < 0.01), body weight (*p* < 0.01), and BMI (*p* = 0.02)) were significantly higher in male patients. No significant differences were observed in preoperative clinical scores between male and female. All clinical scores significantly improved at 2 years postoperatively in both sexes. In the clinical scores, the KOOS-symptom (*p* = 0.03), KOOS-QOL (*p* = 0.03), and Tegner activity scores (*p* < 0.01) showed significantly better scores in male patients.

**Conclusion:**

Following MMPR pullout repair, the clinical outcomes significantly improved in both sexes. These results indicate that MMPR pullout repair is a universally effective technique regardless of the disadvantages of females in morphological characteristics.

**Supplementary Information:**

The online version contains supplementary material available at 10.1007/s00590-025-04344-y.

## Introduction

In human knee structures, the meniscus has an important multifunctional role to maintain overall function of the knee [1]. Among several functions of the meniscus, the most important factor for the prevention of osteoarthritis (OA) is the maintenance of the hoop tension of the medial meniscus (MM) that allows for the correct intra-articular load transmission [1]. Notably, MM posterior root tears (PRTs) lead to the loss of hoop tension, which is the same degree as total meniscectomy [2]. For these reasons, they can cause rapidly progressive knee OA. Nonoperative management of MMPRTs is associated with poor long-term clinical and radiographic outcomes, demonstrating a failure rate as high as 95%, with 53% of patients ultimately undergoing total knee arthroplasty within 14 years [3]. In contrast, MM posterior root (PR) repair has been reported to yield good results clinically and biomechanically, and lead to significantly less OA progression and subsequent knee arthroplasty compared with non-operative management and partial meniscectomy [2, 4].

Common MMPR repair methods include pullout repair of MM posterior attachment by creating a tibial tunnel, and suture anchor fixation of the MMPRT [1]. Recently, MMPR repair combined with centralization using anchors and all-inside suture devices have been reported to provide better short-term clinical outcomes, reduction in the MM extrusion (MME), and restoration of the load-distributing function of the MM [5]. In addition, advances have also been made in methods for assessing meniscal healing, such as the arthroscopic healing score [6]. Thus, many studies have sought to improve the postoperative clinical outcomes of MMPR repair.

Some factors related to poor postoperative outcomes in MMPRTs have been reported. Older age, female sex, high-grade chondral lesions, varus alignment of > 5°, and high body mass index (BMI) are factors associated with poor postoperative outcomes for MMPR repair as well as the occurrence of MMPRT [7, 8]. With regard to postoperative rehabilitation or management, it has been reported that decreased quadriceps muscle strength after MMPR repair was associated with postoperative MME progression [9], and that postoperative weight loss was associated with better meniscal healing and less MME progression after MMPR repair [10], highlighting the significance of quadriceps muscle strength and weight management.

In general, male and female differ in various physical characteristics. Moreover, in the context of MMPRTs, they exhibit differences in morbidity and clinical course. However, to the best of our knowledge, no study has yet compared the postoperative clinical outcomes and investigated anatomical differences between sexes. In this study, we aimed to evaluate the postoperative clinical outcomes and arthroscopic healing status after pullout repair for MMPRTs by sex. We hypothesized that postoperative clinical outcomes and healing status would improve in both males and females, with some significant difference between them.

## Materials and methods

### Patients

This retrospective study was performed according to the Declaration of Helsinki and approved by our institutional review board (approval no. 1857). Written informed consent was obtained from all patients. Between October 2016 and November 2019, a total of 98 patients underwent pullout repair for isolated MMPRTs at our institution. The surgical indications of pullout repair for the symptomatic MMPRTs at our institution were as follows: (1) femorotibial angle (FTA), the external angle of the femoral and tibial shaft axes on coronal radiographs in the standing position, of less than 180°, (2) mild medial compartment OA [Kellgren–Lawrence (KL) grade 0–2] and (3) cartilage lesions classified as grade of 0-II according to the Outerbridge classification system. A total of 12 patients were subsequently excluded: 11 patients with BMI > 30 kg/m^2^ at the initial visit, and 1 patient lost to follow-up. Ultimately, 86 patients followed up for more than 2 years were included in this retrospective survey. No patient was excluded based on age, activity level, or history of ipsilateral knee surgery. The surgery was performed by a single experienced surgeon. The time from injury to surgery was determined through detailed interviews regarding painful popping episodes.

### Surgical technique and second-look arthroscopic evaluation

**Fig. 1:**
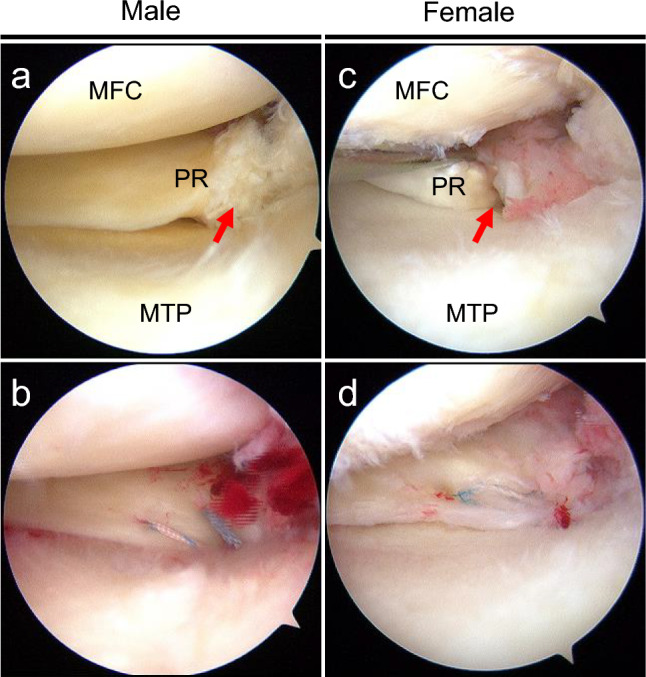
Representative arthroscopic findings of the primary surgery in male (**a** and **b**) and female (**c** and **d**). **a** Medial meniscus (MM) posterior root tear (PRT) was confirmed (red arrow). **b** Pullout repair was performed using two simple stitches concomitant with an additional all-inside suture. **c** MMPRT was confirmed by probing (red arrow). **d** Pullout repair was performed using a modified Mason-Allen suture. MFC, medial femoral condyle; MTP, medial tibial plateau; PR, posterior root (color figure online)

**Fig. 2:**
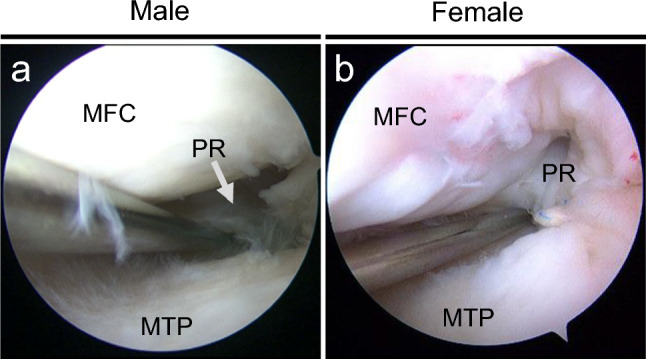
Representative arthroscopic findings of a second-look arthroscopy at 1 year postoperatively in male **a** and female **b**. **a** Sufficient width, stability, and synovial coverage were observed, with scores of 4, 3, and 1, totaling 8. **b** Sufficient width, stability, and synovial coverage were observed, with scores of 4, 4, and 2, totaling 10. MFC, medial femoral condyle; MTP, medial tibial plateau; PR, posterior root

Pullout repair was performed as previously described [11]. The patient was positioned supine with an air tourniquet, and standard anterolateral and anteromedial portals were created. Subsequently, an outside-in pie-crusting technique was performed to facilitate the procedures in the medial knee compartment. MMPRT was confirmed and classified according to the LaPrade classification [12] (Fig. 1). Depending on the time of the surgery, three techniques were used. A modified Mason-Allen suture technique using Ultrabraid and FasT-Fix (F-MMA) all-inside suture (Smith & Nephew, Andover, MA, USA) (n = 25), two simple stitches (TSS) pullout technique using No. 2 polyethylene sutures, such as Ultrabraid and FiberWire (Arthrex, Naples, FL, USA) (n = 15), and TSS pullout technique concomitant with an additional posteromedial all-inside suture (TSS-PM), such as FasT-Fix and AIR (Stryker, Kalamazoo, MI, USA) (n = 46), were performed. Subsequently, a custom-made PRT guide (Smith & Nephew) was used to insert a 2.4 mm diameter guide pin (Smith & Nephew) aiming at the anatomic attachment of the MM posterior root at a 45° angle, followed by overdrilling with a 4.0 mm diameter cannulated drill (Arthrex) to create a tibial tunnel. Following this, the sutures grasping MM were pulled out through the tunnel (Fig. 1). Tibial fixation of the pullout sutures was performed using a bioabsorbable interference screw (Smith & Nephew) at 20–30° of knee flexion with an initial tension of 20–30 N and GTS cancellous screw (Smith & Nephew). Second-look arthroscopic evaluation and screw removal were performed in all patients at 1 year postoperatively. Meniscal healing status was assessed using a semi-quantitative scoring system ranging from 0 to 10 points, with 10 representing complete healing [6] (Fig. 2).

### Postoperative rehabilitation

The rehabilitation protocol consisted of immobilizing the knee joint in extension and a non-weight bearing for 2 weeks. Subsequently, a knee flexion exercise gradually increasing up to 90° under partial weight bearing condition was prescribed. At 6 weeks postoperatively, patients were allowed full weight bearing and 120° of knee flexion. Daily activities accompanied by deep knee flexion and sports activities were allowed after 3 months postoperatively.

### Preoperative and postoperative MRI assessment

The presence of the MMPRT was defined according to characteristic MRI findings such as cleft sign, giraffe neck signs of the MM posterior root within 9 mm from the attachment [1, 13]. Morphological characteristics were assessed using preoperative MRI. Intercondylar notch width (ICNW) and the intercondylar distance (ICD) were measured on coronal MRI slices at the plane of the popliteal groove [14]. The notch width index (NWI) was calculated using the following formula, as previously described: 100 × ICNW/ICD [15]. The MRI-based MM body width (MMBW) and MME were also evaluated both preoperatively and at 2 years postoperatively. The relative MME (r-MME) was calculated as: 100 × MME/MMBW [16]. Postoperative MRI-based MM healing status was assessed at 2 years postoperatively based on the continuity of the MM root and the presence of the suspension bridge sign on coronal images [17].

### Clinical scores

Lysholm scores [18], Tegner activity scores [18], Knee Injury and Osteoarthritis Outcome Score (KOOS) [18], International Knee Documentation Committee (IKDC) scores [18], and pain visual analog scale (VAS) scores [19] were assessed preoperatively and at 2 years postoperatively. The Tegner activity score ranges from 0 to 10, with 10 representing the maximum score, whereas other scores range from 0 to 100, with 100 indicating a perfect score. KOOS consists of five subscales: pain, symptoms, activities of daily living (ADL), sport/recreation function (Sport/Rec), and quality of life (QOL).

### Statistical analysis

Data are presented as the mean ± standard deviation (SD). Statistical analyses were performed using the EZR software (Saitama Medical Center, Saitama, Japan) [20]. Wilcoxon’s signed-rank test was used to compare intragroup difference, and the Mann–Whitney U test was employed to compare intergroup difference in continuous variables. Fisher’s exact test was used to compare intergroup differences in categorical variables. Since KOOS consists of five subscales, analysis was conducted for each subscale. Statistical significance was set at *p* < 0.05.

## Results

**Fig. 3:**
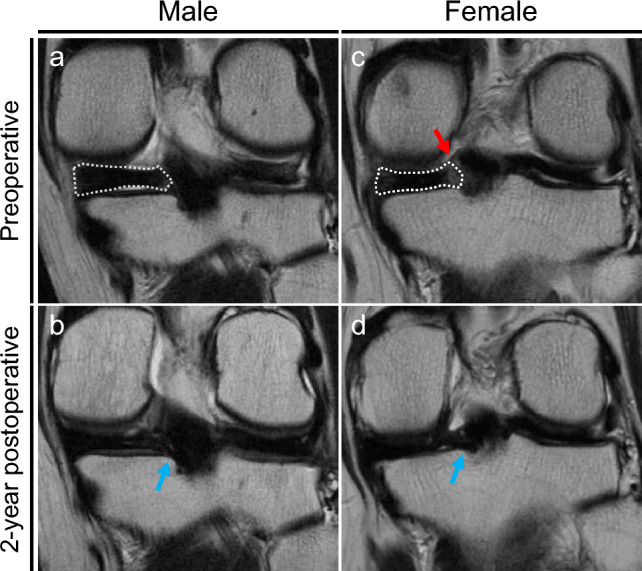
Representative magnetic resonance images (MRI) preoperatively and 2 years postoperatively in male (**a** and **b**) and female (**c** and **d**). **a** Preoperative coronal MRI presents Giraffe neck sign (dotted area). **b** Postoperative coronal MRI shows a continuity of the medial meniscus (blue arrow). **c** Preoperative coronal MRI presents Giraffe neck sign (dotted area) and cleft sign (red arrow). **d** Postoperative coronal MRI shows a continuity of the medial meniscus (blue arrow) (color figure online)

A total of eighty-six patients were included in this study. The demographic data are presented in Table 1. Three factors related to physical status (height (*p* < 0.01), body weight (*p* < 0.01), and BMI (*p* = 0.02)) were significantly higher in male patients. The ICNW was significantly narrower, and the NWI was significantly lower in female patients (Table 1). No significant differences were observed in preoperative clinical scores between male and female (Table 2). Preoperative MRI showed typical findings of MMPRT in both male and female patients (Figs. 3 a and c).

**Table 1:** Demographics and clinical characteristics

	Male	Female	*P* value
Number of patients	21	65	
Age (years)	64.2 ± 9.0	65.6 ± 7.6	0.73
Height (m)	1.70 ± 0.1	1.54 ± 0.1	< 0.01*
Body weight (kg)	72.1 ± 8.9	57.4 ± 7.1	< 0.01*
Body mass index (kg/m^2^)	25.8 ± 2.4	24.2 ± 2.7	0.02*
Femorotibial angle (°)	177.2 ± 1.5	177.4 ± 1.8	0.59
Medial tibial slope (°)	10.0 ± 3.0	9.1 ± 3.1	0.42
Intercondylar notch width (mm)	22.2 ± 1.7	18.3 ± 1.5	< 0.01*
Notch width index (%)	29.6 ± 1.9	28.4 ± 2.1	0.03*

Data of age, height, body weight, body mass index, femorotibial angle, medial tibial slope, intercondylar notch width and notch width index are displayed as a mean ± standard deviation. Notch width index indicates the ratio of the femoral intercondylar notch width to that of the intercondylar distance at the plane of the popliteal groove. Statistical differences between two groups were analyzed using Manne-Whitney U-test. *P < 0.05

**Table 2:** Comparison of preoperative clinical outcomes between male and female

		Male	Female	*P* value
KOOS	Pain	63.1 ± 15.3	58.0 ± 20.1	0.69
	Symptoms	69.0 ± 16.8	63.7 ± 19.3	0.57
	ADL	74.6 ± 14.0	68.1 ± 16.4	0.24
	Sport/Rec	30.0 ± 24.0	26.2 ± 25.5	0.41
	QOL	31.0 ± 16.6	32.0 ± 19.2	0.66
Lysholm score		63.1 ± 10.5	61.0 ± 9.0	0.76
IKDC score		42.9 ± 16.2	37.2 ± 16.1	0.23
Tegner activity score		2.0 ± 1.0	1.6 ± 1.0	0.12
Pain visual analogue scale		39.7 ± 25.9	38.3 ± 26.6	0.97
Preoperative MME (mm)		3.89 ± 1.5	3.75 ± 1.4	0.92
Preoperative r-MME (%)		33.7 ± 16.4	37.3 ± 12.6	0.14

ADL, activities of daily living; IKDC, International Knee Documentation Committee; KOOS, Knee Injury and Osteoarthritis Outcome Score; MME, medial meniscus extrusion; QOL, knee-related quality of life; r-MME, relative medial meniscus extrusion; Sport/Rec, sport and recreation function. Data are displayed as a mean ± standard deviation. Statistical differences between two groups were analyzed using Manne-Whitney U-test. * *P* < 0.05

Meniscal healing status was evaluated at a second-look arthroscopy at 1 year postoperatively (Fig. 2). No significant difference in the total healing score was observed between male and female patients (Table 3).

**Table 3:** Comparison of postoperative clinical outcomes between male and female

		Male	Female	*P* value
KOOS	Pain	90.3 ± 14.1	87.9 ± 14.4	0.48
	Symptoms	90.1 ± 10.1	84.8 ± 12.4	0.03*
	ADL	92.7 ± 11.3	90.1 ± 11.8	0.41
	Sport/Rec	64.1 ± 26.7	60.0 ± 29.9	0.51
	QOL	75.4 ± 19.4	66.4 ± 19.9	0.03*
Lysholm score		90.7 ± 3.7	88.9 ± 7.3	0.39
IKDC score		73.7 ± 16.2	67.0 ± 16.1	0.21
Tegner activity score		3.7 ± 0.8	3.1 ± 0.6	< 0.01*
Pain visual analogue scale		5.8 ± 11.6	9.4 ± 14.5	0.30
Arthroscopic healing score		6.9 ± 2.0	6.8 ± 1.4	0.97
Postoperative MME (mm)		4.84 ± 1.42	5.02 ± 1.74	0.53
Positive rate of suspension bridge sign (%)		63.2	61.4	1

ADL, activities of daily living; IKDC, International Knee Documentation Committee; KOOS, Knee Injury and Osteoarthritis Outcome Score; MME, medial meniscus extrusion; QOL, knee-related quality of life; Sport/Rec, sport and recreation function. Data are displayed as mean ± standard deviation. Arthroscopic healing score at second-look arthroscopy (total, 10 points). Statistical differences in continuous variables/categorical variables between two groups were analyzed using Manne-Whitney U-test/Fischer’s exact test. *P < 0.05

MRI scanned at 2 years postoperatively in both male and female showed a sufficient continuity of the repaired posterior root (Figs. 3 b and d). All clinical scores significantly improved at 2 years postoperatively in both male and female patients (Supplementary Tables 1 and 2). At 2 years postoperatively, the KOOS-symptom (*p* = 0.03), KOOS-QOL (*p* = 0.03), and Tegner activity scores (*p* < 0.01) showed significantly better scores in male compared to female patients (Table 3). However, The changes (Δvalues) in clinical scores between preoperative and postoperative assessments showed no significant differences between male and female patients (Table 4). Additionally, no significant differences were observed in the positive rate of suspension bridge sign (Table 3) or in ΔMME (Table 4).

**Table 4:** Comparison of changes (Δvalues) in clinical outcomes between male and female

		Male	Female	*P* value
KOOS	Pain	27.2 ± 17.3	29.2 ± 20.5	0.96
	Symptoms	21.1 ± 19.4	21.0 ± 17.9	0.58
	ADL	18.1 ± 14.7	21.7 ± 16.1	0.59
	Sport/Rec	34.1 ± 29.9	33.5 ± 35.4	0.84
	QOL	44.4 ± 22.6	34.4 ± 23.4	0.05
Lysholm score		27.6 ± 12.4	29.8 ± 14.8	0.64
IKDC score		30.8 ± 19.2	30.5 ± 18.0	0.77
Tegner activity score		1.67 ± 1.2	1.5 ± 1.0	0.35
Pain visual analogue scale		−33.9 ± 24.0	−27.0 ± 27.9	0.43
ΔMME (mm)		1.27 ± 1.7	0.96 ± 1.1	0.38

ADL, activities of daily living; IKDC, International Knee Documentation Committee; KOOS, Knee Injury and Osteoarthritis Outcome Score; QOL, knee-related quality of life; Sport/Rec, sport and recreation function. Data are displayed as a mean ± standard deviation. ΔMME is a change in medial meniscus extrusion. Statistical differences between two groups were analyzed using Manne-Whitney U-test. *P < 0.05

## Discussion

In this study, the most notable finding was that all clinical scores significantly improved at 2 years postoperatively in both male and female patients. Although some postoperative clinical scores were significantly higher in male compared to female patients, the differences did not reach the Minimal Clinically Important Difference (MCID) threshold [21]. Therefore, our hypothesis was partially supported.

In previous studies, a favorable functional prognosis after pullout repair of MMPRT has been reported. Postoperative clinical outcomes were significantly improved to about 80–85, compared to preoperative Lysholm scores, about 50 [22]. Furthermore, all the postoperative functional scores have been reported to improve and the pain VAS scale to decrease significantly compared to preoperative scores even in three different types of pullout repair techniques of MMPR repair (F-MMA, TSS, and TSS-PM) [23]. The postoperative clinical outcomes for both male and female patients were comparable to those of previous reports. These findings suggest that MMPR repair is an effective surgical treatment for both male and female individuals.

However, at present, factors related to good postoperative outcomes have yet to be sufficiently analyzed. Increasing age, increased BMI, preoperative MME, and varus degree have been reported as risk factors for poor clinical outcomes following MMPR repair [7]. Among these, patients with a high BMI were excluded from this study. The Lysholm score improved from 63.1 ± 10.5 to 90.7 ± 3.7 in male patients (n = 21, *p* < 0.01) and from 61.0 ± 9.0 to 88.9 ± 7.3 in female patients (n = 65, *p* < 0.01) at a mean follow-up of 2 years. The fact that favorable postoperative scores were obtained in both male and female patients in this study suggests that BMI may be a major factor in poor prognosis after MMPR repair.

In previous reports, differences in the anatomical and physical characteristics between male and female patients have been reported. Females have been shown to have increased posterior tibial and meniscal slopes, narrower femoral notches, smaller native ligaments, and a higher proportion of joint surface sliding with consecutive anterior tibial translation compared with male [24, 25]. A previous study reported that a narrower ICNW is associated with poorer clinical outcomes following MMPR repair [15]. In this study, although the ICNW was significantly narrower in female patients, Δclinical scores and ΔMME were comparable between male and female patients. These findings suggest that MMPR pullout repair is also effective in female patients, despite potential disadvantages related to morphological characteristics.

This study had several limitations. First, the sample size was relatively small. Second, the initial tension and knee flexion for tibial fixation of the pullout sutures differed between 20–30N and 20–30°. Third, bone mineral density were not evaluated in patients included in this study. Finally, other factors not verified in this study, including differences of muscle strength between male and female patients, may be confounding factors in addition to sex differences.

## Conclusion

This study demonstrated that pullout repair for MMPRT significantly improved clinical outcomes in both male and female patients, when patients with high BMI were excluded. These results indicated that this surgical technique is a universally effective technique regardless of the disadvantages of females in morphological characteristics.

### Clinical relevance

This study demonstrated that pullout repair for MMPRT improved clinical outcomes in both male and female patients.

## Supplementary Information

Below is the link to the electronic supplementary material.

Supplementary file1 (DOCX 18 KB)

## Data Availability

The datasets generated and analyzed during the current study are available from the corresponding author on reasonable request.
