# Supplementary material for: Favorable clinical outcomes are achieved in both male and female following medial meniscus posterior root repair
Source: Eur J Orthop Surg Traumatol. 2025 Jun 14;35(1):245. doi: 10.1007/s00590-025-04344-y (PMC12167242; doi:10.1007/s00590-025-04344-y)
Supplement: Supplementary file 1 — Supplementary file1 (DOCX 18 KB) [file 590_2025_4344_MOESM1_ESM.docx]

**Favorable clinical outcomes are achieved in both male and female following medial meniscus posterior root repair**

**Supplementary Information**

Supplementary table 1. Comparison between pre- and postoperative clinical outcomes in male

|  |  | Preoperative | Postoperative | P value |
| --- | --- | --- | --- | --- |
| KOOS | Pain | 63.1 ± 15.3 | 90.3 ± 14.1 | <0.01* |
|  | Symptoms | 69.0 ± 16.8 | 90.1 ± 10.1 | <0.01* |
|  | ADL | 74.6 ± 14.0 | 92.7 ± 11.3 | <0.01* |
|  | Sport/Rec | 30.0 ± 24.0 | 64.1 ± 26.7 | <0.01* |
|  | QOL | 31.0 ± 16.6 | 75.4 ± 19.4 | <0.01* |
| Lysholm score | | 63.1 ± 10.5 | 90.7 ± 3.7 | <0.01* |
| IKDC score | | 42.9 ± 16.2 | 73.7 ± 16.2 | <0.01* |
| Tegner activity score | | 2.0 ± 1.0 | 3.7 ± 0.8 | <0.01* |
| Pain visual analogue scale | | 39.7 ± 25.9 | 5.8 ± 11.6 | <0.01* |

KOOS, Knee Injury and Osteoarthritis Outcome Score; IKDC, International Knee Documentation Committee; ADL, activities of daily living; Sport/Rec, sport and recreation function; QOL, knee-related quality of life. Data are displayed as a mean ± standard deviation. Statistical differences between two groups were analyzed using Wilcoxon signed-rank sum test. * P < 0.05

Supplementary table 2. Comparison between pre- and postoperative clinical outcomes in female

|  |  | Preoperative | Postoperative | P value |
| --- | --- | --- | --- | --- |
| KOOS | Pain | 58.0 ± 20.1 | 87.9 ± 14.4 | <0.01* |
|  | Symptoms | 63.7 ± 19.3 | 84.8 ± 12.4 | <0.01* |
|  | ADL | 68.1 ± 16.4 | 90.1 ± 11.8 | <0.01* |
|  | Sport/Rec | 26.2 ± 25.5 | 60.0 ± 29.9 | <0.01* |
|  | QOL | 32.0 ± 19.2 | 66.4 ± 19.9 | <0.01* |
| Lysholm score | | 61.0 ± 9.0 | 88.9 ± 7.3 | <0.01* |
| IKDC score | | 37.2 ± 16.1 | 67.0 ± 16.1 | <0.01* |
| Tegner activity score | | 1.6 ± 1.0 | 3.1 ± 0.6 | <0.01* |
| Pain visual analogue scale | | 38.3 ± 26.6 | 9.4 ± 14.5 | <0.01* |

KOOS, Knee Injury and Osteoarthritis Outcome Score; IKDC, International Knee Documentation Committee; ADL, activities of daily living; Sport/Rec, sport and recreation function; QOL, knee-related quality of life. Data are displayed as a mean ± standard deviation. Statistical differences between two groups were analyzed using Wilcoxon signed-rank sum test. * P < 0.05
